# Genome-Wide Screen of Genes Required for Caffeine Tolerance in Fission Yeast

**DOI:** 10.1371/journal.pone.0006619

**Published:** 2009-08-12

**Authors:** Isabel A. Calvo, Natalia Gabrielli, Iván Iglesias-Baena, Sarela García-Santamarina, Kwang-Lae Hoe, Dong Uk Kim, Miriam Sansó, Alice Zuin, Pilar Pérez, José Ayté, Elena Hidalgo

**Affiliations:** 1 Oxidative Stress and Cell Cycle Group, Universitat Pompeu Fabra, Barcelona, Spain; 2 Pombe Deletion Project, KRIBB, Yuseong-gu, Daejeon, Republic of Korea; 3 Instituto de Microbiología Bioquímica, CSIC, Salamanca, Spain; National Institutes of Health (NIH)/National Institute of Environmental Health Sciences (NIEHS), United States of America

## Abstract

**Background:**

An excess of caffeine is cytotoxic to all eukaryotic cell types. We aim to study how cells become tolerant to a toxic dose of this drug, and the relationship between caffeine and oxidative stress pathways.

**Methodology/Principal Findings:**

We searched for *Schizosaccharomyces pombe* mutants with inhibited growth on caffeine-containing plates. We screened a collection of 2,700 haploid mutant cells, of which 98 were sensitive to caffeine. The genes mutated in these sensitive clones were involved in a number of cellular roles including the H_2_O_2_-induced Pap1 and Sty1 stress pathways, the integrity and calcineurin pathways, cell morphology and chromatin remodeling. We have investigated the role of the oxidative stress pathways in sensing and promoting survival to caffeine. The Pap1 and the Sty1 pathways are both required for normal tolerance to caffeine, but only the Sty1 pathway is activated by the drug. Cells lacking Pap1 are sensitive to caffeine due to the decreased expression of the efflux pump Hba2. Indeed, *?hba2* cells are sensitive to caffeine, and constitutive activation of the Pap1 pathway enhances resistance to caffeine in an Hba2-dependent manner.

**Conclusions/Significance:**

With our caffeine-sensitive, genome-wide screen of an *S. pombe* deletion collection, we have demonstrated the importance of some oxidative stress pathway components on wild-type tolerance to the drug.

## Introduction

The methylxanthine derivative caffeine is an analogue of purine bases which has been involved in a variety of cellular processes in eukaryotic cells, including mammals, plants and fungi. Caffeine has shown a wide array of pharmacological and biological effects that interfere with DNA repair and recombination pathways, delay cell cycle progression and modulate intracellular calcium homeostasis. However, the manner in which caffeine triggers these pleiotropic effects is still largely unknown. Many groups have used genetically tractable organisms to study the biological and toxic effects of caffeine. Thus, in *Saccharomyces cerevisiae*, caffeine has been reported to affect cell cycle progression [1], [2] and cell morphology and integrity [3]. In *Schizosaccharomyces pombe*, caffeine has been demonstrated to inhibit repair mechanisms [4], [5], and to interfere with both meiotic [6] and UV-induced mitotic [7] recombination. Furthermore, caffeine is known to be an inhibitor of cAMP phosphodiesterase in different eukaryotic cell types [8].

Most of the reports which use unicellular eukaryotes to unravel the effects of caffeine are based on the isolation of strains which display enhanced resistance to cytotoxic levels of the drug, either by a chromosomal mutation [9], [10] or by over-expression from a multicopy plasmid [11]. In order to become tolerant to a toxic drug, over-expression or modification of a target molecule would allow cells to withstand a higher concentration. Also, amplification of repair or scavenger activities could improve survival. Finally, cells with altered import (reduced) or export (increased) of the drug would display higher tolerance to caffeine.

In *S. pombe*, the Sipiczki laboratory isolated a number of caffeine-resistant mutants which defined single loci. Thus, the *caf1–21*, *caf2–3*, *caf3–89*, *caf4–83* and *caf5* mutants displayed pleiotropic, albeit slightly different, phenotypes to the cells: caffeine resistance, increased sensitivity to UV-irradiation, a reduction in fertility, lengthening of the cell cycle and some morphological aberrations [10]. These common features suggested that all the *caf* genes had related functions and define a single caffeine-responsive “tolerance” pathway. Indeed, all five mutations have finally been reported to be connected to the Pap1 pathway. Pap1 is an AP1-like transcription factor with cytosolic localization prior to stress; moderate doses of hydrogen peroxide (H_2_O_2_) trigger oxidation of Pap1 and its fast accumulation in the nucleus, with the concomitant activation of an adaptive antioxidant response [12], [13]. Further studies enabled the identification of the genes which were altered in the *caf* mutants [11], [14]. Thus, *caf2–3* carries a loss-of-function mutation at *crm1*; Crm1 is the nuclear exporter of Pap1, and the defective *caf2–3* allele leads to constitutive nuclear localization of Pap1 [9], [14]. Hba1 is a cofactor of the Crm1-mediated export of Pap1, and the *caf1–21* mutation contains an early stop codon in the open-reading frame [15]. *caf3–89* has a gain-of-function of the *pap1* gene: the encoded Pap1 protein has constitutive nuclear localization [11]. *caf4–83* carries a loss of function mutation at *trr1*; the lack of the thioredoxin reductase Trr1 leads to the constitutive oxidation and therefore nuclear localization of Pap1 [13]. Lastly, over-expression of the *caf5* locus mutation has been described to enhance the protein levels of an ABC transporter, Caf5 [11]; the expression of this transporter is dependent on Pap1 [16].

Using microbes as model systems, several groups have isolated genes related to oxidative stress pathways in the search for mutants with increased resistance to unrelated drugs (multidrug resistant phenotype). This could be due to a natural induction of the stress pathway by the drugs, since they could trigger reactive oxygen species production. Alternatively, many oxidative stress regulons include ATP-binding cassette (ABC)-family transporters among the genes induced upon stress, which may act as efflux pumps to extrude the drugs from the intracellular compartment. In *S. pombe*, there are two alternative oxidative stress pathways; the Pap1-dependent one responds to moderate concentrations of H_2_O_2_, and the MAP kinase Sty1 pathway becomes activated not only upon toxic doses of H_2_O_2_ but also in response to heat shock, osmotic stress and other situations which compromise cell viability (for a review, see [17]). Only over-expression of the Pap1, but not Sty1, pathway has arisen as beneficial in overcoming high doses of caffeine [9], [10].

We decided to initiate an alternative approach to gain insights into the molecular targets of caffeine, and to study in depth its relationship with oxidative stress pathways. We searched for mutants from *S. pombe* with inhibited growth on caffeine-containing agar plates, using a deletion collection of about 2,700 haploid mutant cells, of which 98 were sensitive to the drug. The genes mutated in these sensitive clones were involved in a number of cellular roles including the stress, the integrity and the calcineurin pathways. Also, genes involved in the establishment of cell morphology, chromatin remodeling and protein traffic were identified as essential to maintain a wild-type tolerance to caffeine. We confirmed the sensitivity of most clones by sequential dilutions on solid plates, and investigated the role of the Pap1 and Sty1 stress pathways with regard to caffeine toxicity.

## Results

### Genome-wide screen of caffeine-sensitive mutants

**Figure 1:**
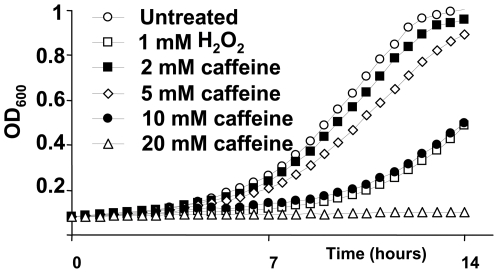
Growth curves of wild-type *S. pombe* in the presence or absence of caffeine or H_2_O_2_. Log-phase cultures at an OD_600_ of 0.1 of the wild-type strain 972 were treated or not with the indicated concentrations of caffeine or H_2_O_2_, and grown into microculture wells. Growth was monitored by measuring OD_600_ every 10 min at 30° for 14 h.

Different concentrations of caffeine in liquid media can either partially or completely inhibit the growth of *S. pombe*, in a similar way as H_2_O_2_ does (Figure 1) [18]. A comparable cytotoxic effect can be observed when caffeine is added to solid plates. In order to paint a global picture of the cellular mechanisms used by *S. pombe* to cope with toxic doses of caffeine, we carried out a genome-wide isolation of mutants displaying growth defects in the presence of 10 mM caffeine. We spread a collection of about 2,700 haploid mutants, and searched for cells with impaired growth on YE plates containing the drug. We obtained 98 putative isolates. The sensitivity to caffeine of 59 of those strains was confirmed by sequential spotting (see below).

With our screen, a number of mutants were isolated whose sensitivity to caffeine had already been established, but many other were new. We grouped the mutants by functional categories, and analyzed each one of them by sequential spots on solid plates containing caffeine (see Materials and Methods). Since most mutations enhancing resistance to the drug had been described as leading to constitutive activation of the oxidative stress-dependent Pap1 pathway (see Introduction), we also plated our putative caffeine-sensitive strains in H_2_O_2_-containing plates. Only those mutants confirmed to be sensitive to caffeine by sequential spotting are shown in Supplementary Table S1. We have also included in this table some *S. pombe* mutants that were not positive in the initial screen (either because the genome-wide plating was less sensitive than the sequential spots, or because those particular mutants were not present in the deletion collection), but were subsequently checked (and came up as positives) in the spot assays. Among the strains isolated in the initial screen, both *Δsty1* and *Δpap1* cells displayed a strong sensitive phenotype, and were used thereafter, together with a wild-type strain, as controls in all the spot assays we performed.

### Involvement of the Pap1 pathway in cellular tolerance to caffeine

**Figure 2:**
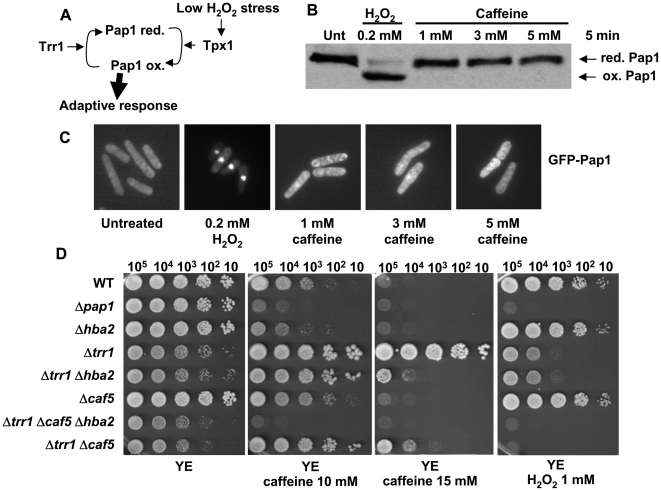
Pap1 is not activated by caffeine, but is required for normal tolerance to caffeine. (A) Scheme of the physiological activation of the transcription factor Pap1 by extracellular peroxides. The role of Tpx1 and Trr1 in activation and inactivation, respectively, of Pap1 are indicated. (B) Pap1 is not oxidized by caffeine *in vivo*. Wild-type strain 972 was grown in minimal media and treated or not with H_2_O_2_ or caffeine at the indicated concentrations, during 5 minutes. The redox state of Pap1 was analyzed by Western blot after non-reducing electrophoresis. Reduced/inactive (red.Pap1) and oxidized/active (ox.Pap1) Pap1 forms are indicated with arrows. (C) Pap1 is not accumulated in the nucleus upon caffeine treatment. The cellular distribution of GFP-Pap1 was determined by fluorescence microscopy in strain EHH14, treated or not with H_2_O_2_ or caffeine at the indicated concentrations, during 5 minutes. (D) Survival to caffeine or H_2_O_2_ exposure in YE media plates of strains deleted on components of the Pap1 pathway. Strains 972 (WT), AV25 (*Δpap1*), NG28 (*Δhba2*), NG24 (*Δtrr1*), NG42 (*Δtrr1 Δhba2*), NG29 (*Δcaf5*), NG37 (*Δtrr1 Δcaf5 Δhba2*), and NG39 (*Δtrr1 Δcaf5*) were grown in liquid YE media, and the indicated number of cells were spotted onto plates with or without caffeine or H_2_O_2_, at the indicated concentrations.

Deletion of the gene coding for the Pap1 transcription factor rendered cells sensitive to caffeine (Figure 2D). Pap1, which becomes oxidized by moderate doses of H_2_O_2_ and thereafter accumulates at the nucleus to trigger an anti-oxidant gene response (Figure 2A) [13], did not become oxidized (Figure 2B) nor did it accumulate at the nucleus (Figure 2C) by any dose of caffeine we tested. Thus, the *pap1* gene is essential for normal tolerance to caffeine, and other proteins related to the pathway, such as the Pap1-regulated Srx1 and Trx1, are essential as well (Supplementary Figure S1). Conversely, activation of the pathway, e.g. by deletion of the thioredoxin reductase Trr1, enhances the resistance to caffeine (Figure 2D), as described earlier [14].

Since AP1-like transcription factors have been described to promote caffeine resistance by up-regulating efflux pumps [19], we analyzed whether lack of the ABC-transporter Hba2/Bfr1 and/or Caf5, whose genes are under the control of the transcription factor Pap1 [20], would abolish the caffeine-resistant phenotype of *Δtrr1* cells. Indeed, *Δtrr1* cells lacking both *hba2* and *caf5* genes were very sensitive to caffeine (Figure 2D). In fact, cells lacking Hba2 were almost as sensitive to caffeine as *Δpap1* cells (Figure 2D), indicating that Hba2 is the major caffeine exporter in *S. pombe.* It has been reported, and we have confirmed it by Northern blot analysis (data not shown), that *hba2* basal transcription is 3-fold lower in *Δpap1* than in wild-type cells [20], which explains the sensitivity to caffeine of cells lacking Pap1.

### Activation of the MAP kinase Sty1 stress pathway by caffeine

**Figure 3:**
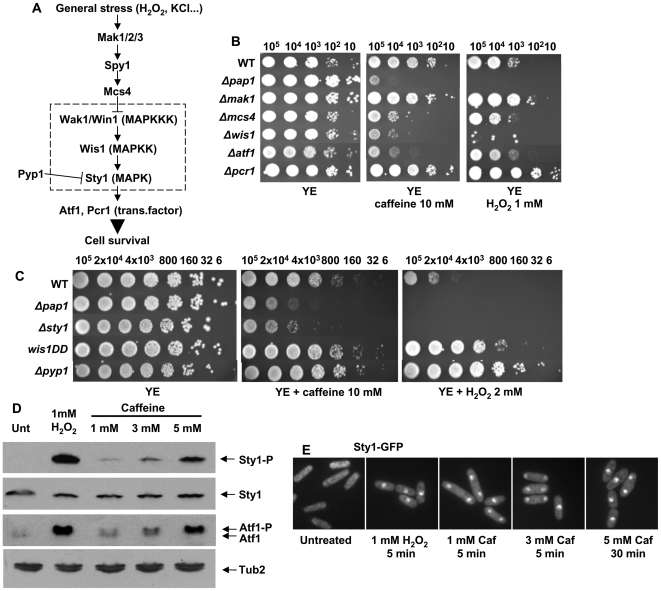
Sty1 is activated by caffeine, and is required for normal tolerance to caffeine. (A) Scheme of the activation of the MAP kinase Sty1 by extracellular stressors. Other upstream and downstream components of the pathway are indicated. (B, C) Survival to caffeine or H_2_O_2_ of strains harboring mutations in components of the Sty1 pathway. We analyzed by sequential spotting (as described in Figure 2D) the survival of strains 972 (WT), AV25 (*Δpap1*), AV15 (*Δatf1*), KS2088 (*wis1DD*), and the deletion collection strains *Δmak1*, *Δmcs4*, *Δwis1*, *Δpcr1*, and *Δpyp1*. (D) Sty1 is phosphorylated and Atf1 is activated in response to caffeine. The levels of Sty1 phosphorylation (Sty1-P) in the wild-type strain 972 grown in minimal medium, which had been treated or not with caffeine or H_2_O_2_, were determined by Western blot analysis using anti-phosphorylated p38 antibody. The same blots were hybridized with polyclonal anti-Atf1. The slower-migrating phosphorylated (Atf1-P) and the non-phosphorylated (Atf1) forms are indicated with arrows. Polyclonal antibodies against Sty1 and tubulin (Tub2) were used as loading controls. (E) Sty1-GFP is accumulated in the nucleus upon caffeine treatment. The cellular distribution of Sty1-GFP in the strain EHH5 grown in minimal medium, and treated or not with H_2_O_2_ or caffeine (Caf) for the times indicated was determined by fluorescence microscopy.

Components of the general stress pathway, centered on the MAP kinase Sty1 (Figure 3A), were sensitive to caffeine and to H_2_O_2_ (Figure 3B). Pcr1, a b-ZIP transcription factor known to heterodimerize with Atf1 to induce the Sty1-dependent gene response, is dispensable for the cellular response to caffeine (Figure 3B), as described above for other stresses [21]. It is worth pointing out that the screens performed by others to isolate genes whose mutations increased resistance to multidrugs had never brought up components of the Sty1 pathway. Consistently, we tested that constitutive activation of the pathway, either by expression of a constitutively activated MAP kinase kinase Wis1DD or by deletion of the MAP kinase phosphatase Pyp1, enhanced resistance to toxic doses of H_2_O_2_ but did not increase or just slightly improved the tolerance to caffeine (Figure 3C).

The Sty1 pathway is activated in response to a whole variety of stress signals (for a review, see [17]). We determined that caffeine also induced phosphorylation of the Sty1 MAP kinase (Figure 3D), which triggered a rapid translocation of Sty1-GFP from the cytosol to the nucleus (Figure 3E) and the phosphorylation and accumulation of its main substrate, the transcription factor Atf1 (Figure 3D). Therefore, caffeine activates the main global anti-stress response pathway known to be required for survival upon compromised environmental situations.

**Figure 4:**
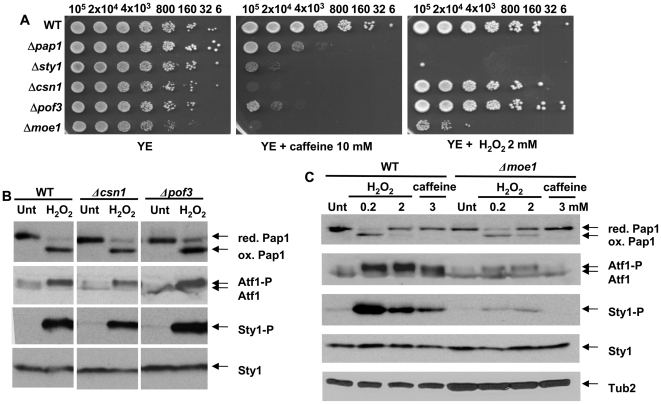
Regulators of protein stability are required for normal tolerance to caffeine. (A) Survival to caffeine or H_2_O_2_ exposure at the indicated concentrations of strains harboring mutations in genes coding for regulators of protein homeostasis. We analyzed by sequential spotting as described in Fig. 2D the survival of strains 666 (WT), and the deletion collection strains *Δpap1*, *Δsty1*, *Δcsn1*, *Δpof3* and *Δmoe1*. (B) Csn1 and Pof3 do not regulate the Pap1 or Sty1 pathways. Wild-type strain 666 (WT) and the deletion collection strains *Δcsn1* and *Δpof3* were grown in minimal media. TCA extracts were prepared from treated (5 min 0.2 mM H_2_O_2_) or untreated (Unt) cultures, and the redox state of Pap1, and phosphorylation levels of Sty1 and Atf1 were determined as described in Fig. 2B and Fig. 3D. (C) Moe1 regulates Atf1 protein levels and Sty1 phosphorylation. Strains 666 (WT) and *Δmoe1* were grown in minimal media, and were treated or not (Unt) for 5 min with H_2_O_2_ or caffeine at the indicated concentrations. TCA extracts and immunoblot assay was performed as described in B. Sty1 total protein (Sty1) and tubulin (Tub2) were used as loading controls.

Once established that both stress pathways, Pap1 and Sty1, are essential to maintain normal sensitivity to caffeine, we centered our attention on three genes isolated in our screen which regulate protein levels, and which may therefore exert their effects through regulation of Pap1, Sty1 or its main transcription factor, Atf1: *moe1* (may regulate translation or protein stability) [22], [23], *csn1* (involved in the signalosome) [24] and *pof3* (F-box protein, may specifically regulate protein levels) [25]. As shown in figure 4A, only deletion of *moe1* confers sensitivity to both caffeine and H_2_O_2_. Concomitantly, only cells lacking Moe1 have altered levels of Atf1, and display a deficient activation of Sty1 (Figure 4B and 4C). The participation of the Moe1-interacting partner Int6 in wild-type tolerance to caffeine and its relationship with Atf1 protein levels has been recently assessed [26], [27]. Further work will be required to determine the role of Moe1 in Sty1 phosphorylation.

### Other cellular processes

**Figure 5:**
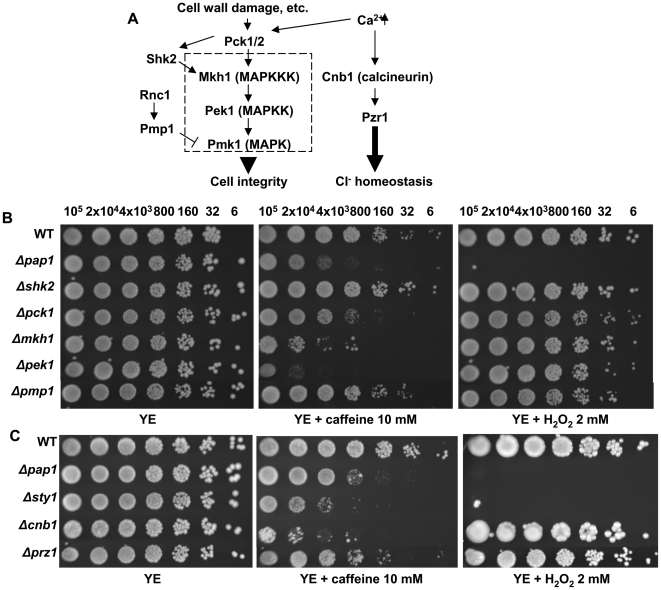
The cell integrity and the calcineurin pathways are required for normal tolerance to caffeine. (A) Scheme of the activation of the MAP kinase Pmk1 by cell wall damage. Other upstream and downstream components of the pathway are indicated. The calcineurin components are also indicated. (B, C) Survival to caffeine or H_2_O_2_ exposure at the indicated concentrations of strains harboring mutations in genes coding for components of the Pmk1 (B) or Cnb1 (C) pathways. Survival of the deletion collection strains 666 (WT), *Δpap1*, *Δshk2*, *Δpck1*, *Δmkh1*, *Δpek1*, *Δpmp1*, *Δsty1*, *Δcnb1* and *Δprz1* was analyzed by sequential spotting, as described in Fig. 2D.

In our screen we isolated other mutations in genes coding for activities related to processes previously connected to caffeine, and that validates the results obtained. Thus, we found that several strains carrying deletions in genes coding for proteins of the cell integrity pathway whose central component is the MAP kinase Pmk1 (Figure 5A) were sensitive to caffeine, as described earlier [28], [29], but not to H_2_O_2_ (Figure 5B). Similarly, strains carrying deletions in genes involved in cell polarity, cell wall biosynthesis or cytokinesis, which are related to the cell integrity pathway, are also over-represented (Supplementary Figure S2).

A role for caffeine in the regulation of *S. cerevisiae* calcium homeostasis has been described earlier [30]. Mutations in components of the calcineurin pathway also led to caffeine sensitivity in our screen (Figure 5C) suggesting that caffeine also inhibits extracellular Ca^2+^ uptake in fission yeast.

**Figure 6:**
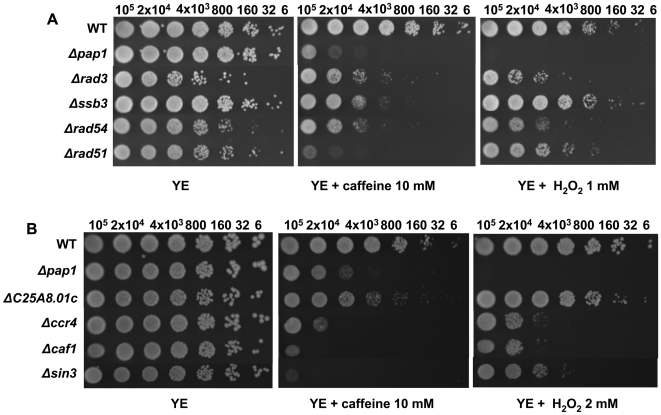
Several regulators of chromatin remodeling (A) and DNA repair/recombination pathways (B) are required for normal tolerance to caffeine. We analyzed by sequential spotting as described in Fig. 2D the survival to caffeine or H_2_O_2_ exposure at the indicated concentrations of the deletion collection strains 666 (WT), *Δpap1*, *Δrad3*, *Δssb3*, *Δrad54*, *Δrad51, ΔC25A8.01c*, *Δccr4*, *Δcaf1* and *Δsin3*.

Other cell functions linked to caffeine toxicity have been impaired recombination and DNA damaging [31]. In agreement with those studies, several deletions in genes coding for activities related to recombination and/or repair have been isolated in the screen, such as *rad3*, *ssb3*, *rad54* and *rad51* (Figure 6A). Similarly, intracellular protein traffic is one of the major functional categories, with many gene deletions conferring lower tolerance to caffeine (vacuole protein sorting, Golgi or ER function, etc.) (Supplementary Figure S3). Probably this traffic is required to eliminate the caffeine. On the other hand, a pathway traditionally linked to caffeine tolerance is the protein kinase A. However, the sensitivity to caffeine of *S. pombe* cells lacking Pka1 was only slightly higher, if any, of that of a wild-type strain (Supplementary Figure S4). Furthermore, strains bearing mutations in other components of the pathway did not display any growth inhibition by caffeine (Supplementary Figure S4).

Other genes isolated in the screen and that therefore have functions related to the cell response to caffeine might be indirectly affecting pathways required to counteract the effects of caffeine or required to facilitate its degradation. Thus, general regulators of mRNA abundance, such as the chromatin remodeler *SPAC25A8.01c*, members of the Ccr4 complex, or the histone acethyl transferase Sin3 are present in this global list (Figure 6B). Several genes related to general metabolic pathways also altered the tolerance to caffeine (Supplementary Figure S5), as well as genes known to regulate the meiotic or mitotic cell cycles (Supplementary Figure S6).

## Discussion

**Figure 7:**
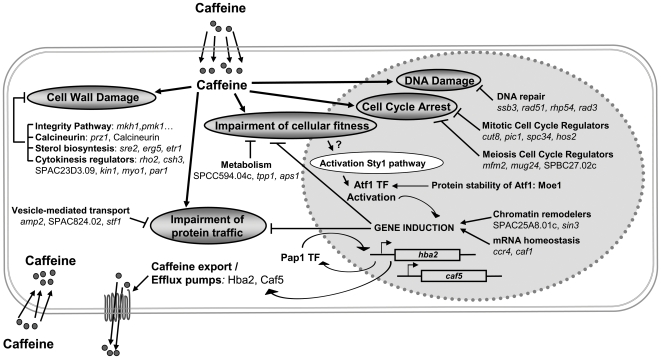
Proposed model for the cellular targets and defense response mechanisms to caffeine in fission yeast. The main detoxification mechanism for caffeine is extrusion by efflux pumps Hba2 and Caf5, which basal expression levels are dependent on the transcription factor (TF) Pap1. Pap1 is not activated by caffeine, but it shuttles under basal conditions between the nucleus (in pale grey, dotted line) and the cytosol, and regulates the basal expression of about 50 genes [20]. Caffeine interferes with cellular activities and induces a number of toxic effects (in grey ovals): cell wall damage, protein traffic and general fitness impairment, cell cycle arrest and DNA damage. Most of the genes we have isolated in our screen code for proteins which can combat each one of those deleterious effects of caffeine, and have been grouped accordingly. The Sty1 pathway is activated in response to caffeine through an unknown mechanism. Activated Sty1 is then essential to activate changes in gene expression through the transcription factor Atf1, and these changes may modulate the cellular adaptation to caffeine. Chromatin remodelers and regulation of mRNA homeostasis may help regulate gene induction in response to caffeine as well. Lastly, proteins such as Moe1 may be essential to wild-type tolerance to caffeine due to its role in the regulation of Atf1 protein basal levels.

Caffeine, which elicits well-documented cytotoxic effects to eukaryotic cells, has been proposed to target and inactivate many cellular activities (Fig. 7). Several genetic approaches had been undertaken to identify those targets, frequently based on the isolation of caffeine-resistant microbial cells, and very often constitutive activation of oxidative stress pathways had been connected to caffeine tolerance. With our caffeine-sensitive, genome-wide screen of an *S. pombe* deletion collection, we have demonstrated the importance of some oxidative stress pathway components on wild-type tolerance to the drug. Furthermore, we have demonstrated with a parallel screen on H_2_O_2_-containing plates that some, but not all, of the caffeine-sensitive mutants also display defects in the presence of H_2_O_2_. Thus, cells lacking components of the Pap1 and Sty1 pathways, the intracellular protein transport system, cell polarity machinery, DNA recombination/repair systems, and chromatin remodelling regulators are both sensitive to caffeine and to H_2_O_2_ (Supplementary Table S1).

We do not, however, believe that any of the toxic effects of caffeine is mediated through direct generation of reactive oxygen species, since the sensitive Pap1 pathway is not induced at any concentration of the drug (we have tested caffeine concentrations ranging from 0.05 to 30 mM, and none of them activate Pap1; data not shown). The global stress response pathway, centered on the MAP kinase Sty1, does become activated by caffeine. However, this pathway is not only triggered by H_2_O_2_, but also by any type of environmental stress which compromises cell viability, and caffeine does so. Up-regulation of the Sty1 pathway had never been isolated as a genetic component of resistance to caffeine, and that is consistent with our results: inactivation of the pathway by the deletion of some components increases sensitivity to the drug, but hyper-activation of the pathway through the lack of the Sty1 phosphatase Pyp1, or through expression of a constitutively active Wis1 kinase, does not significantly enhance the tolerance to caffeine.

In contrast, lack of Pap1 triggers sensitivity and up-regulation of Pap1 induces resistance to caffeine. We show here that such an effect is mainly due to a downstream target of Pap1, the gene coding for the efflux pump Hba2. The development of multidrug resistance in microorganisms may be due to a number of mechanisms. The most documented one is enhanced extrusion of drugs mediated by efflux pump proteins belonging to either the ABC (ATP-binding cassette) or MFS (major facilitator) superfamilies; these efflux pumps are able to extrude structurally diverse compounds. The abundance of the drug transporters and their wider specificity suggest that they may not be exclusively drug exporters in microbes and may have other cellular functions. In some cases, their expression levels are regulated by environmental signals; that is the case of the oxidative-stress dependent *acrAB* locus of *Escherichia coli* [32], the *mexGHI-ompD* four-gene operon of *Pseudomonas aeruginosa*, which encodes a multidrug efflux pump system involved in quorum-sensing signal homeostasis and which may be activated by superoxide [33], or the export pumps for glutathione S-conjugates, which have been cloned from mammals, yeast, plants, and nematodes (for a review, see [34]). In the case of the Pap1 regulon, up-regulation of the pathway increases the expression of both Hba2 and Caf5 [20], and these efflux pumps induce a multidrug resistant phenotype. Our data indicate that Hba2 is the major efflux pump for caffeine, since deletion of its gene causes sensitivity to the drug (Figure 2D). However, Caf5 is also able to partially extrude caffeine, since the triple *Δtrr1 Δhba2 Δcaf5* strain displays stronger sensitivity to the drug that *Δtrr1 Δhba2* cells (Figure 2D). It is worth noting that over-expression of the Pap1 homolog YAP1 of *S. cerevisiae* also confers resistance to several drugs [35], and that such a phenotype is dependent on the presence of two efflux pumps, FLR1 and YCF1, whose expression is under the control of YAP1 [36]. However, deletion of the *YAP1* gene does not result in sensitivity to cycloheximide [37] or diazaborine [36], indicating that either YAP1 in *S. cerevisiae* is not such a strong determinant of multidrug resistance as Pap1 is in *S. pombe*, or that the basal levels of *FLR1/YCF1* transcripts are unchanged in *ΔYAP1* cells (*hba2* basal transcription is 3-fold lower in *Δpap1* cells than in wild-type cells) [20].

The demonstration of linkage between a gene deletion and a phenotype is only a first step that might unveil details of a whole cellular response to an environmental stress. With our screen, we have further explored additional cellular pathways involved in caffeine resistance and we have identified genes belonging to pathways participating in *S. cerevisiae* survival to caffeine (Fig. 7). These genes validate our screen and corroborate the biological significance of conserved processes between the two distant yeasts. Thus, it was not surprising to isolate genes coding for the cell integrity MAP kinase pathway (Figure 5A & B) as well as cell morphology genes related to that pathway (Supplementary Figure S2). For several microorganisms, caffeine is currently used as a phenotypic criterion to evaluate the function of cell wall integrity pathways [38]. Similarly, it has been described that *S. cerevisiae* uptake of the extracellular Ca^2+^ is inhibited by caffeine [30], and, according to our results, that is probably the case in fission yeast (Figure 5C).

It was also predictable to find intracellular protein traffic as one of the major functional categories, with many caffeine-sensitive gene deletions (Supplementary Figure S3). Caffeine acts as a competitive inhibitor for adenosine and its presence likely causes an artificial metabolic stress to the cells. Probably traffic to the vacuole is required to eliminate the caffeine in *S. pombe* as in *S. cerevisiae* [39]. Additionally, some metabolic pathways might be required to counteract the caffeine competitive inhibition effect.

Caffeine was the first drug reported to override checkpoints and several reports described caffeine inhibition of Rad3 [31], and Rad-related kinases ATM or ATR in mammalian cells [40]–[42]. Importantly enough, three genes known to be involved in replication, recombination and/or repair (*rad3, rad51* and *rhp54*) were isolated as essential for normal tolerance to both caffeine and H_2_O_2_ (Figure 6A), highlighting the importance of DNA homeostasis in the response to both insults.

A surprising result from our screen concerns the cAMP-signalling pathway traditionally involved in caffeine tolerance, with cAMP phosphodiesterase being perhaps the best known protein target inactivated by the drug [8]. However, we have not detected a significant alteration of tolerance to caffeine in any of the mutants of this pathway that we have tested (Supplementary Figure S4). Similarly, the TORC1 kinase has recently been described as the growth-limiting target of caffeine [43], [44], but the homologous Tor2 kinase is essential and therefore its deletion mutant was not present in the collection, and other components of the pathway were not isolated in our screen. These results suggest, but do not demonstrate, that *S. pombe* cAMP phosphodiesterase is not an essential caffeine target while other signalling pathways important to *S. pombe* survival are affected by this drug.

## Materials and Methods

### Yeast strains and growth conditions

We used the strains 972 (*h* ^−^), JA364 (*h^+^ ura4-D18*), JA365 (*h^−^ ura4-D18*), AV18 (*h* ^−^ *sty1*::*kanMX6*) [45], AV25 (*h* ^−^ *pap1*::*kanMX6*) [45], EHH14 (*h^+^ his2 ura4-D18 pap1*::*ura4-D18 leu1–32 nmt*::*GFP-pap1*::*leu1* ^+^) [46], KS2088 (*h^−^ ura4-D18 wis1DD::12myc::ura4^+^ leu1–32 sty1::HA6H::ura4^+^*) [47], AV15 (*h^−^ atf1::kanMX6)* [45], EA38 (*h^−^ leu1–32 srx1::kanMX6*) [48] and EHH5 (*h^−^ leu1–32 sty1*::*GFP*::*kanMX6*) [45]. To construct *S. pombe* strains with specific loci deleted, we transformed wild-type strains (either 972 or JA364) with linear fragments containing open reading frame *(ORF)::kanMX6* or *ORF::natMX6*, obtained by PCR amplification using ORF-specific primers and plasmids pFA6a-kanMX6 [49] or pFA6a-natMX6 [50] as templates, and we obtained strains NG28 (*h^+^ hba2*::*natMX6*), NG29 (*h^−^ caf5*::*kanMX6*), MJ2 (*h^−^ trx1*::*kanMX6 ura4-D18 leu1–32*), NG35 (*h^+^ hba2::natMX6 ura4-D18*), NG34 (*h^+^ hba2::natMX6 caf5::kanMX6 ura4-D18*) and NG41 (*h^−^ caf5:: kanMX6 ura4-D18*). NG24 (*h^−^ caf4^+^::ura4^+^ ura4-D18* ) was isolated after crossing *Δcaf4* (*h^90^ caf4^+^::ura4^+^ ura4-D18 ade6–704 leu1–32*) [14] with JA365 (*h^−^ ura4-D18*). To obtain NG42 (*h^−^ hba2::natMX6 caf4::ura4^+^ ura4-D18*), we crossed NG35 with NG24. NG37 (*h^+^ hba2::natMX6 caf5::kanMX6 caf4^+^::ura4* ^+^ *ura4-D18*) was isolated after crossing the double mutant NG34 with NG24. We isolated NG25 (*h^+^ caf4^+^::ura4^+^ ura4-D18)* after crossing NG24 with JA364. To obtain NG39 (*h^+^ caf5:: kanMX6 caf4::ura4* ^+^ *ura4-D18*) we crossed NG41 *w*ith NG25. Cells were grown in standard media [minimal media or rich media (YE)] [51], with or without caffeine or H_2_O_2_ at the indicated concentrations.

### Growth curves

To measure cellular growth we used an assay based on automatic measurements of optical densities (OD) of small (100 µl) liquid cell cultures, which allowed us to plot comparable growth curves for each treatment. Basically, we grew cells in YE media to an OD_600_ of 0.3 at 30°C under continuous shaking in Erlenmeyer flasks. Then, we diluted the cultures in YE media to an OD_600_ of 0.025 and cells continued growing in the same conditions till they reached an OD_600_ of 0.1. We treated the cultures with different agents (2, 5, 10 and 20 mM caffeine and 1 mM H_2_O_2_). Then, we placed 100-µl samples into 96-well non-coated polystyrene microplates (in triplicate) with an adhesive plate seal. We used Power Wave microplate scanning spectrophotometer (Bio-Tek) to obtain the growth curves. The OD_600_ was automatically recorded using Gen5 software. The software was set as follows: OD was measured at 600 nm, incubation temperature was kept at 30°C, the microplates were subjected to continuous shaking and the readings were done every 10 min during a 14 h period.

### High-throughput sensitivity screen

Genome-wide *S. pombe* haploid deletion collection covers more than 2,700 genes. *S. pombe* diploid deletion mutants were systematically constructed with targeted mutagenesis at each ORF, and haploid deletion strains for non-essential genes were isolated. The wild-type strains of the collection are 666 (*h^+^ ade6-M210 ura4-D18 leu1–32*) and 668 (*h^+^ ade6-M216 ura4-D18 leu1–32*). More information is provided at the Bioneer web page (http://pombe.bioneer.co.kr/introduction/ResearchPurpose.jsp). The haploid deletion collection was screened as described elsewhere [52]. Basically, the collection was first grown in liquid YE media, and then spread with a manual replicator on three types of solid agar plates: YE media, YE media with 5 mM H_2_O_2_ and YE media with 10 mM caffeine. The plates were incubated at 30°C for 3*–*4 days.

### Caffeine and H_2_O_2_ sensitivity assay by sequential spots

In order to carefully analyze sensitivity to caffeine on plates, *S. pombe* strains were grown in liquid YE media to an OD_600_ of 0.5. Cells were then diluted in YE, and the indicated number of cells in 2 µl was spotted onto YE media agar plates, containing or not the indicated concentrations of caffeine (10 or 15 mM) or H_2_O_2_ (1 or 2 mM). Plates were incubated at 30°C for 3*–*4 days.

### Preparation of *S. pombe* TCA extracts and immunoblot analysis

To analyze the *in vivo* redox state of Pap1, trichloroacetic acid (TCA) extracts were prepared as described elsewhere [48]. Immunoblotting was performed as described [45]. Pap1 was immunodetected using polyclonal anti-Pap1 antibody [13]. A different protocol to obtained TCA extracts (without alkylation nor phosphatase treatment) was followed to detect Atf1, and has been described elsewhere [21]. Same extracts were prepared to detect phosphorylated and non-phosphorylated Sty1. Immunoblotting was performed using a commercial anti-p38 MAP kinase antibody (Cell Signalling), or polyclonal anti-Sty1 antibodies raised against bacterial glutathione-S-transferase (GST)-Sty1 following standard rabbit immunization procedures. As a loading control, monoclonal anti-tubulin antibody (Tub2, Sigma) was used.

### Fluorescence microscopy

Fluorescence microscopy and image capture was performed as described before [13].

## Supporting Information

Table S1 (0.16 MB PDF)

Figure S1 Several proteins related to the Pap1 pathway are required for normal tolerance to caffeine. We analyzed by sequential spotting (as described in Fig. 2D) the survival to caffeine or H_2_O_2_ exposure at the indicated concentrations of MJ2 (*Δtrx1*, coding for the cytosolic thioredoxin), EA38 (*Δsrx1*, coding for the Tpx1 reductase Srx1); and the deletion collection strains 666 (WT), *Δpap1, Δsty1* and *Δtrx2* (coding for the mitochondrial thioredoxin).
(3.53 MB TIF)

Figure S2 Several regulators of cell polarity or cell wall biosynthesis are required for normal tolerance to caffeine. We analyzed by sequential spotting (as described in Fig. 2D) the survival to caffeine or H_2_O_2_ exposure at the indicated concentrations of the deletion collection strains 666 (WT), *Δpap1, Δsty1, Δmyo1, ΔC306.06c, Δpar1, ΔC23D3.09, Δkin1, Δrho2*, and *Δcsh3*.
(6.71 MB TIF)

Figure S3 Several components of intracellular protein sorting are required for normal tolerance to caffeine. We analyzed by sequential spotting (as described in Fig. 2D) the survival to caffeine or H_2_O_2_ exposure at the indicated concentrations of the deletion collection strains 666 (WT), *Δpap1, Δsty1, Δvps32, ΔC4B3.02C, Δdid4, ΔC613.01, Δryh1, Δerd2, Δsec28*, and *Δsft1*.
(6.59 MB TIF)

Figure S4 The protein kinase A pathway is not required for normal tolerance to caffeine. We analyzed by sequential spotting (as described in Fig. 2D) the survival to caffeine or H_2_O_2_ exposure at the indicated concentrations of the deletion collection strains 666 (WT), *Δpap1, Δsty1, Δcgs2, Δgit3, Δpka1, Δfbp1, Δcgs1, Δsck2* and *Δrsv1*.
(3.77 MB TIF)

Figure S5 Several genes coding for enzymes related to metabolic pathways (A) and for mitochondrial components (B) are required for normal tolerance to caffeine. We analyzed by sequential spotting (as described in Fig. 2D) the survival to caffeine or H_2_O_2_ exposure at the indicated concentrations of the deletion collection strains 666 (WT), *Δpap1, Δtpp1, ΔC1778.03c, ΔC594.04c, Δsty1, ΔC2G2.13c, Δsib1, Δsib2, Δaps1, Δerg5, Δcoq2, Δetr1, ΔC20G8* and *Δcoq10*.
(6.58 MB TIF)

Figure S6 Several regulators of the mitotic or meiotic cell cycles are required for normal tolerance to caffeine. We analyzed by sequential spotting (as described in Fig. 2D) the survival to caffeine or H_2_O_2_ exposure at the indicated concentrations of the deletion collection strains 666 (WT), *Δpap1, Δsty1, Δmfm2, Δcut8, Δspc34, Δpic1, Δhos2, ΔC27.02c* and *Δmug24*.
(4.31 MB TIF)
