# Supplementary material for: Genome-Wide Screen of Genes Required for Caffeine Tolerance in Fission Yeast
Source: PLoS One. 2009 Aug 12;4(8):e6619. doi: 10.1371/journal.pone.0006619 (PMC2720375; doi:10.1371/journal.pone.0006619)
Supplement: Table S1 — (0.16 MB PDF) [file pone.0006619.s001.pdf]

Supplementary Table SI. List of *S. pombe* mutants with altered sensitivity to caffeine

Systematic Name	Common name and description	Sensitivity to caffeine in screen	Sensitivity to caffeine by spots	Sensitivity to H_2O_2 by spots
**Stress pathway**				
SPAC1834.08	**mak1,** putative sensory transduction histidine kinase required upstream of Sty1	No	Yes(mild)Fig.3	No-Fig.3
SPAC24B11.06c	**sty1**, serine/threonine protein kinase, Sty1 SAPK cascade,	Yes	Yes-Fig.3	Yes-Fig.3
SPBC887.10	**mcs4**, response regulator receiver domain, involved in response to stress, Sty1 cascade	Yes	Yes-Fig.3	Yes-Fig.3
SPBC409.07c	**wis1**, serine/threonine protein kinase, MAP kinase kinase (MAPKK), Sty1 cascade	Yes	Yes-Fig.3	Yes-Fig.3
SPAC26F1.10c	**pyp1,** protein-tyrosine phosphatase that acts on Sty1p and negatively regulates mitosis	No	Resis. Fig.3	Resis. Fig.3
SPBC29B5.01	**atf1,** cAMP-dependent transcription factor, substrate of Sty1; strain AV15 (lab strain)	No	Yes-Fig.3	Yes(mild)-Fig.3
SPAC21E11.03c	**pcr1,** S. pombe bZIP-containing transcription factor, heterodimerizes with Atf1	No	No-Fig.3	No-Fig.3
**Oxidative stress Pap1 pathway**				
SPAC1783.07c	**pap1,** AP-1-like transcription factor required for oxidative stress responses	Yes	Yes-Fig.2	Yes-Fig.2
SPBC3F6.03	**trr1,** thioredoxin reductase	Not in collec.	Resis-Fig.2	Yes-Fig.2
SPAC7D4.07c	**trx1,** cytosolic thioredoxin Trx1	Not in collec.	Yes-Fig.S1	Yes-Fig.S1
SPBC12D12.07c	**trx2,** mitochondrial thioredoxin Trx2	No	Resis-Fig.S1	No-Fig.S1
SPBC106.02c	**srx1,** sulfiredoxin, reduces hyperoxidized Tpx1; strain EA38 (lab. strain)	Not in collec.	Yes-Fig.S1	Yes-Fig.S1
**Regulation of translation / protein stability /protein modifiers**				
SPAC637.07	**moe1**, eukaryotic translation initiation factor eIF3d	Yes	Yes-Fig.4	Yes-Fig.4
SPBC215.03c	**csn1**, COP9/signalosome complex (subunit 1), required for the removal of Ned8p from Pcu1p, involved in DNA damage checkpoint	Yes	Yes-Fig.4	No-Fig.4
SPCC338.16	**pof3**, F-box protein, TPR repeat protein. Belongs to the SCF ubiquitin ligase complex.	Yes	Yes-Fig.4	No-Fig.4
SPAC1782.05	**rrd2** is involved in rapamycin sensitivity and cell cycle progression, similar to phosphotyrosyl phosphatase activator. PPIases accelerate the folding of proteins.	Pink	Yes	No
SPAC1834.05	**alg9,** glycosyl transferase family 22, , involved in N-linked glycosylation (predicted)	Yes	Yes (mild)	No
**Integrity pathway / calcineurin pathway**				
SPBC119.08	**pmk1**, MAP kinase, involved in cell wall biosynthesis and cell morphogenesis	Yes	Yes	No

SPAC1F3.02c	**mkh1**, MAP kinase kinase, involved in cell wall biosynthesis and cell morphogenesis	Yes	Yes-Fig.5	No-Fig.5
SPBC1685.01	**pmp1,** dual-specificity MAP kinase phosphatase Pmp1	No	No-Fig.5	No-Fig.5
SPCC757.09c	**rnc1**, RNA-binding protein, interacts physically with pmp1 mRNA	Yes	No	No
SPAC1F5.09c	**shk2,** PAK-related kinase Shk2	No	No-Fig.5	No-Fig.5
SPAC17G8.14c	**pck1,** protein kinase C (PKC)-like Pck1	No	Yes-Fig.5	No-Fig.5
SPBC543.07	**pek1,** MAP kinase kinase Pek1	No	Yes-Fig.5	No-Fig.5
SPCC830.06	**calcineurin (b subunit)** (regulatory subunit), protein phosphatase regulatory subunit	Yes	Yes-Fig.5	No-Fig.5
SPAC4G8.13c	**prz1**, transcription factor, dephosphorylated by calcineurin, regulator of pmc1	Yes	Yes-Fig.5	No-Fig.5
**PKA pathway**				
SPBC106.10	**pka1,** cAMP-dependent protein kinase (catalytic subunit), serine/threonine protein kinase, involved in cAMP-mediated signaling, involved in response to nutrients	Yes	Yes (mild)-Fig.S4	Yes (mild)-Fig.S4
**Vesicle-mediated transport**				
SPAC1142.07c	**vps32**, involved in intracellular protein transport, in late endosome to vacuole transport	Yes	Yes-Fig.S3	No-Fig.S3
SPBC4B4.06	**vps25**,DUF852, conserved protein,involved in protein-vacuolar targeting (predicted)	Yes	Yes (mild)	Yes (mild)
SPAC4F8.01	**did4**, involved in intracellular protein transport, in late endosome to vacuole transport	Yes	Yes-Fig.S3	No-Fig.S3
SPAC11H11.01	sequence orphan, coiled-coil (predicted) (region).ESCRT I complex subunit Vps23.	Yes	Yes (mild)	Yes
SPBC651.11c	**apm2**, AP adaptor complex (predicted), involved in vesicle-mediated transport	Yes	Yes (mild)	Yes (mild)
SPCC4B3.02c	involved in intracellular protein transport, involved in ER to Golgi transport (predicted)	Yes	Yes-Fig.S3	Resis-Fig.S3
SPAC824.02	**esterase/lipase**, involved in vesicle-mediated transport, localization ER membrane	Yes	Yes (mild)	Yes (mild)
SPBP8B7.22	**erd2**, HDEL sequence binding, involved in retrograde (Golgi to ER) transport	Yes	Yes-Fig.S3	No-Fig.S3
SPBC24C6.05	**sec28**, subunit of coatomer, reversibly associates with Golgi non- clathrin-coated vesicles	Yes	Yes-Fig.S3	No-Fig.S3
SPAC31A2.13c	**sft1**, SNARE-associated, involved in secretory pathway and in vesicle-mediated transport	Yes	Yes-Fig.S3	No-Fig.S3
SPCC613.01	**transporter**, unknown specificity, localization membrane	Yes	Yes-Fig.S3	No-Fig.S3
SPAC4C5.02c	**ryh1**, small GTPase, deletion mutant is temperature sensitive for growth and sensitive to osmotic stress. Role in retrograde traffricking of proteins	Yes	Yes-Fig.S3	No-Fig.S3
**Cell Polarity / cell wall biosynthesis / cytokinesis**				
SPAC16.01	**rho2**, GTPase, Rho family, involved in cellular morphogenesis, septation and cell	Yes	Yes-Fig.S2	No-Fig.S2

	polarity			
SPBC119.05c	**csh3**, src (SH3) homology domain. Actin cytoskeleton organization and biogenesis	Yes	Yes-Fig.S2	Yes(mild)-Fig.S2
SPAC664.02c	**actin-like protein**, similar to S. cerevisiae YOR141C	Yes	Yes	No
SPAC23D3.09	**actin-like protein**, similar to S. cerevisiae YJL081C	Yes	Yes-Fig.S2	Yes-Fig.S2
SPBC4F6.06	**kin1**, ser/thr protein kinase, microtubule affinity-regulating kinase, involved in cell polarity	Yes	Yes-Fig.S2	Yes(mild)-Fig.S2
SPBC146.13c	**myo1**, myosin (type I), actin cortical patch component, involved in cytokinesis	Yes	Yes-Fig.S2	Yes-Fig.S2
SPCC306.06c	localization ER membrane, involved in cell wall biosynthesis	Yes	Yes-Fig.S2	Yes-Fig.S2
SPCC188.02	**par1**, protein phosphatase regulatory subunit, involved in cytokinesis, cellular morphogenesis and response to stress	Yes	Yes-Fig.S2	Yes(mild)-Fig.S2
**Transcription factors / chromatin remodeling / mRNA homeostasis**				
SPAC144.02	**transcription factor**, zinc finger protein, zf-C2H2 type, no apparent orthologs	Yes	Yes (mild)	No
SPBC354.05c	**sre2**, involved in transcriptional regulation of anaerobically induced genes	Yes	Yes (mild)	No
SPBC3B9.09	**zinc finger protein**, zf-RBZ, similar to S. cerevisiae YLR417W	Yes	Resistant	No
SPAC3G9.04	**ssu72**, phosphoprotein phosphatase activity, TFIIB interacting protein	Yes	Yes (mild)	No
SPAC25A8.01c	**SNF2 family**, helicase C-terminal domain, involved in chromatin remodelling	Pink	Yes-Fig.6	No-Fig.6
SPAC29A4.20	**Sin3;** RNA polymerase II holoenzyme component, histone acetyltransferase	Yes	Yes-Fig.6	Yes-Fig.6
SPCC31H12.08c	**ccr4**, CCR4-Not complex (predicted), transcription initiation	Yes	Yes-Fig.6	Yes-Fig.6
SPCC18.06c	CCR4-Not complex (**caf1**), is required for complete poliA removal from mRNA.	Yes	Yes-Fig.6	Yes-Fig.6
SPBC21B10.03c	**involved in polyadenylation**, similar to S. cerevisiae YGR178C	Yes	Yes (mild)	No
**DNA repair**				
SPCC23B6.05c	**ssb3**, DNA replication factor A (subunit 3), role in DNA recombination, replication, repair	Yes	Yes-Fig.6	No-Fig.6
SPAC644.14c	**rad51**, rhp51, RecA family, required for recombination and for the repair of DNA damage	Yes	Yes-Fig.6	Yes-Fig.6
SPAC15A10.03c	**rhp54**, **rad54.** Role in the processing of replication structures, also in DNA repair	Yes	Yes-Fig.6	Yes-Fig.6
SPBC216.05	**rad3,** ATR checkpoint kinase	Not in collec.	Yes(mild)Fig.6	Yes-Fig.6
**Metabolism**				
SPAC4A8.06c	**esterase/lipase**	Yes	Yes (mild)	No
SPCC594.04c	5 predicted transmembrane helices, steroid metabolic process	Yes	Yes(mild)Fig.S5	No-Fig.S5

SPBC1778.03c	NADH pyrophosphatase, Nudix family hydrolase, involved in NADH metabolism	Yes	Yes(mild)Fig.S5	No-Fig.S5
SPAC19G12.15c	**tpp1**, trehalose-6-phosphate phosphatase, involved in response to stress	Yes	Yes-Fig.S5	No-Fig.S5
SPBC2G2.13c	**deoxycytidylate deaminase** (predicted), similar to S. cerevisiae YHR144C	Yes	Yes-Fig.S5	Yes(mild)Fig.S5
SPAC23G3.02c	**sib1**, peptide synthetase, phosphopantetheine attachment site (3)	Yes?	No-Fig.S5	No-Fig.S5
SPAC23G3.03	**sib2,**peptide monooxygenase	No	Yes(mild)Fig.S5	No-Fig.S5
SPBC15D4.15	**pho2,** 4-nitrophenylphosphatase	Yes	Yes (mild)	No
SPAC13G6.14	**aps1,** diphosphoinositol-polyphosphate diphosphatase activity, dual function MutT hydrolase, involved in inositol polyphosphate hydrolysis	Yes	Yes-Fig.S5	Yes (mild)-Fig.S5
Mitochondrial				
SPAC56F8.04c	**coq2**, para-hydroxybenzoate--polyprenyltransferase, ppt1	Yes	No-Fig.S5	Yes(mild)-Fig.S5
SPAC19A8.04	**erg5**, erg5, C-22 sterol desaturase (predicted), cytochrome p450 (predicted)	Yes	Yes-Fig.S5	No-Fig.S5
SPAC26F1.04c	**etr1**, involved in mitochondrial synthesis of fatty acids	Yes	Yes-Fig.S5	No-Fig.S5
SPAC20G8.04c	electron transfer flavoprotein-ubiquinone oxidoreductase, 4Fe-4S	Yes	No-Fig.S5	No- Fig.S5
SPCC16A11.07	**coq10,** Electron transport and cellular respiration.	Pink	No-Fig.S5	Yes-Fig.S5
**Meiosis**				
SPAC513.03	**mfm2**, M-factor precursor, pheromone, no apparent orthologs	Pink	Yes-Fig.S6	No-Fig.S6
SPCC74.09	**mug24**, protein containing three RNA recognition motifs. Expressed in meiosis	Yes	Yes-Fig.S6	No-Fig.S6
**Mitotic Cell Cycle / chromosome segregation**				
SPAC17C9.13c	**cut8**, essential, involved in anaphase, controls localization of 26S proteasome	Yes	Yes-Fig.S6	No-Fig.S6
SPBC336.15	**pic1**, INCENP-like. Has a role in sister chromatid cohesion and condensation	Yes	Yes-Fig.S6	No-Fig.S6
SPAC8C9.17c	**spc34**, DASH complex, localization spindle pole body, involved in chromosome segregation	Pink	Yes-Fig.S6	No-Fig.S6
SPAC1805.07c	**hos2**, DASH complex, localization spindle pole body, involved in chromosome segregation	Yes	Yes-Fig.S6	No-Fig.S6
SPBC27.02c	**DASH complex,** localization spindle pole body, involved in chromosome segregation	Yes	Yes-Fig.S6	No-Fig.S6
**Sequence Orphan**				
SPBC428.04	sequence orphan, non-essential, 2 predicted transmembrane helices	Yes	Yes (mild)	No
SPBC3H7.12.	sequence orphan	Yes	Yes (mild)	Yes (mild)
