# Supplementary figures and images for: Genome-Wide Screen of Genes Required for Caffeine Tolerance in Fission Yeast
Source: PLoS One. 2009 Aug 12;4(8):e6619. doi: 10.1371/journal.pone.0006619 (PMC2720375; doi:10.1371/journal.pone.0006619)

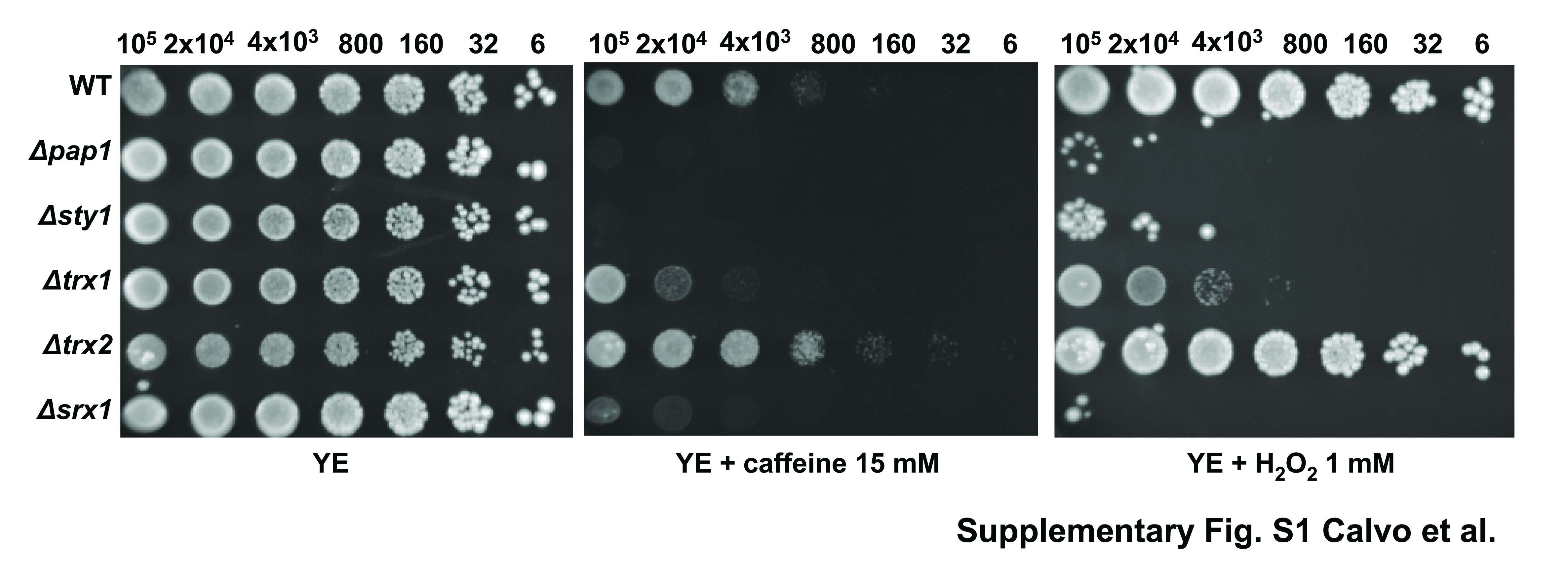

Supplement: Figure S1 — Several proteins related to the Pap1 pathway are required for normal tolerance to caffeine. We analyzed by sequential spotting (as described in Fig. 2D) the survival to caffeine or H2O2 exposure at the indicated concentrations of MJ2 (Δtrx1, coding for the cytosolic thioredoxin), EA38 (Δsrx1, coding for the Tpx1 reductase Srx1); and the deletion collection strains 666 (WT), Δpap1, Δsty1 and Δtrx2 (coding for the mitochondrial thioredoxin). (3.53 MB TIF) [file pone.0006619.s002.tif]

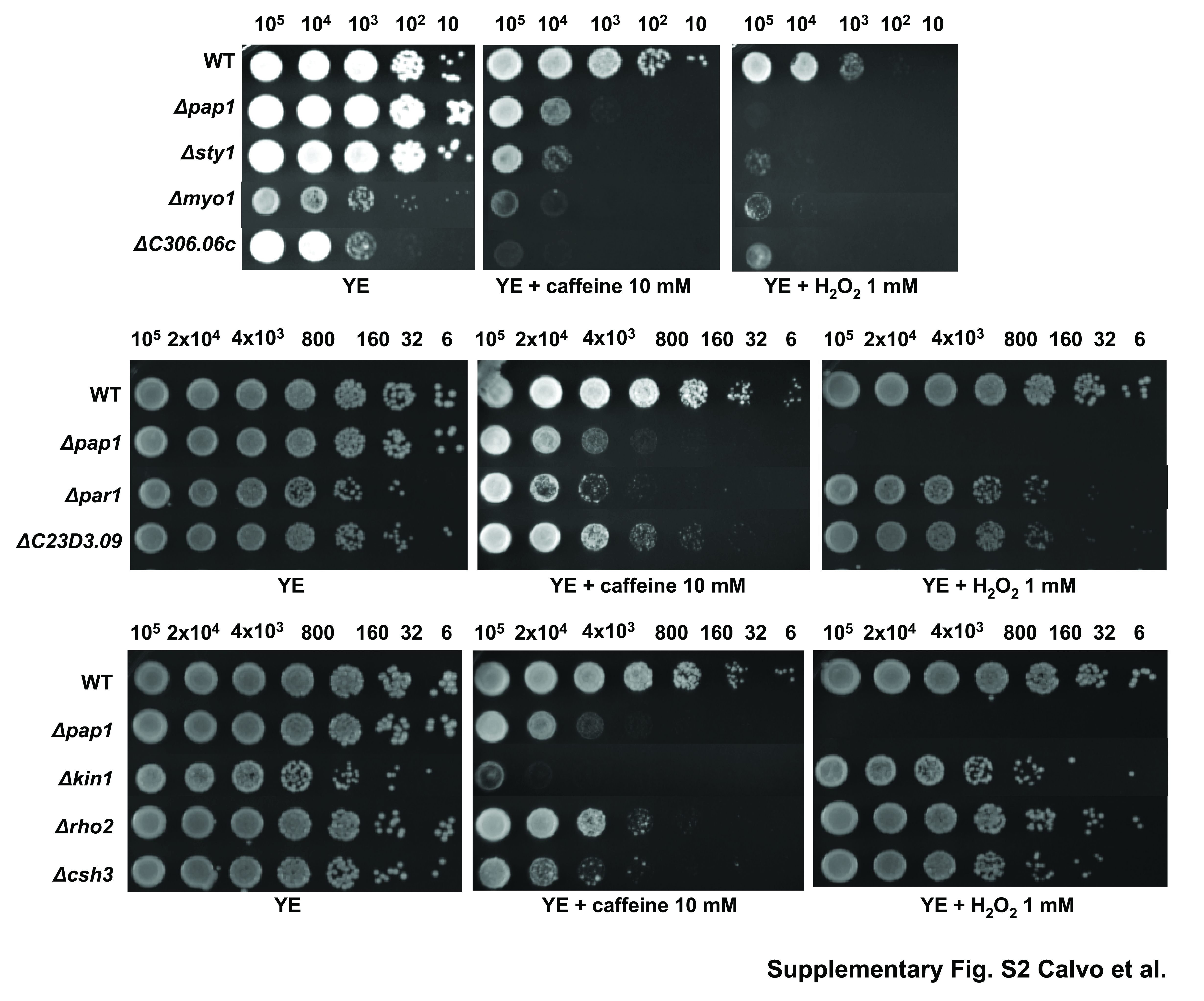

Supplement: Figure S2 — Several regulators of cell polarity or cell wall biosynthesis are required for normal tolerance to caffeine. We analyzed by sequential spotting (as described in Fig. 2D) the survival to caffeine or H2O2 exposure at the indicated concentrations of the deletion collection strains 666 (WT), Δpap1, Δsty1, Δmyo1, ΔC306.06c, Δpar1, ΔC23D3.09, Δkin1, Δrho2, and Δcsh3. (6.71 MB TIF) [file pone.0006619.s003.tif]

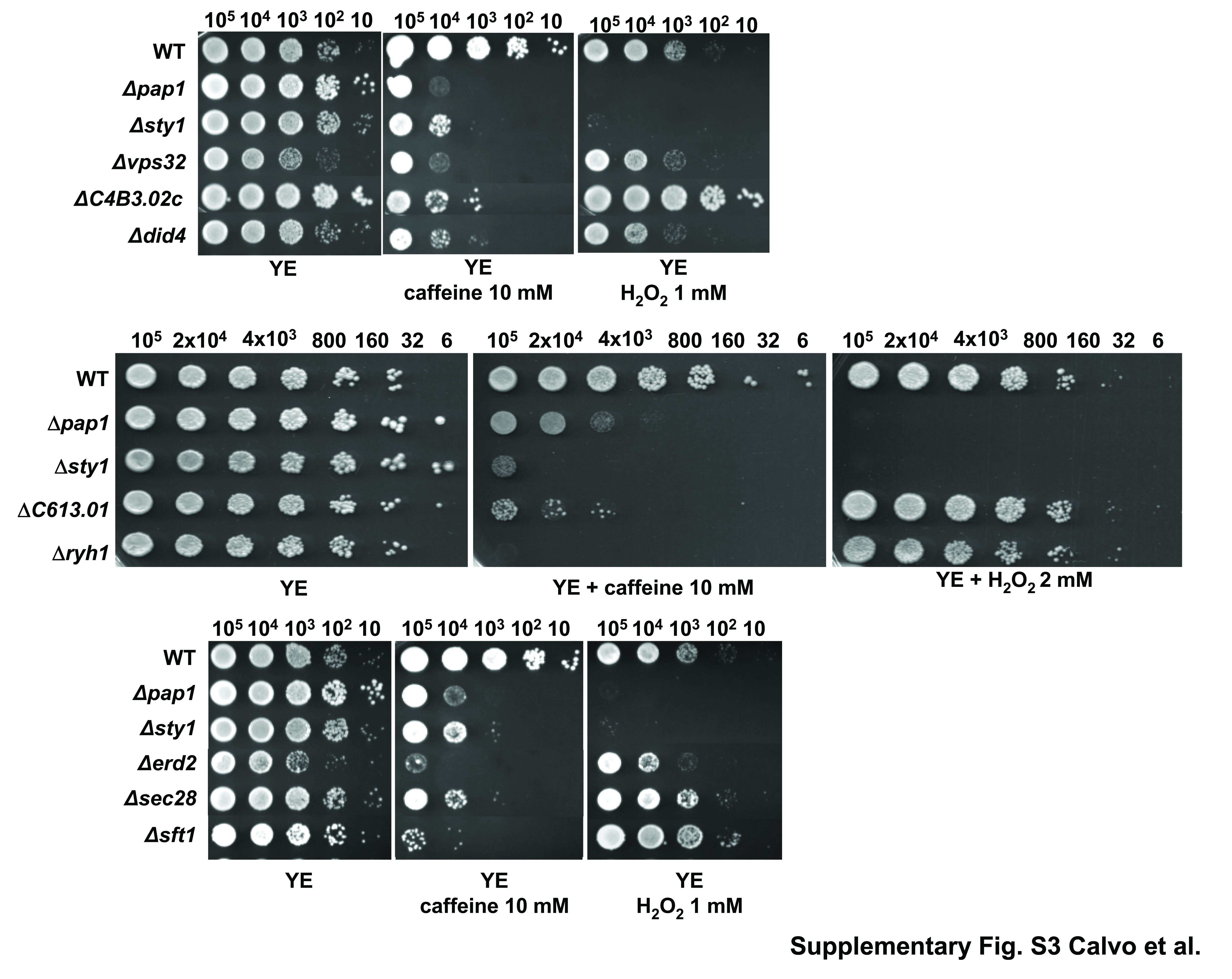

Supplement: Figure S3 — Several components of intracellular protein sorting are required for normal tolerance to caffeine. We analyzed by sequential spotting (as described in Fig. 2D) the survival to caffeine or H2O2 exposure at the indicated concentrations of the deletion collection strains 666 (WT), Δpap1, Δsty1, Δvps32, ΔC4B3.02C, Δdid4, ΔC613.01, Δryh1, Δerd2, Δsec28, and Δsft1. (6.59 MB TIF) [file pone.0006619.s004.tif]

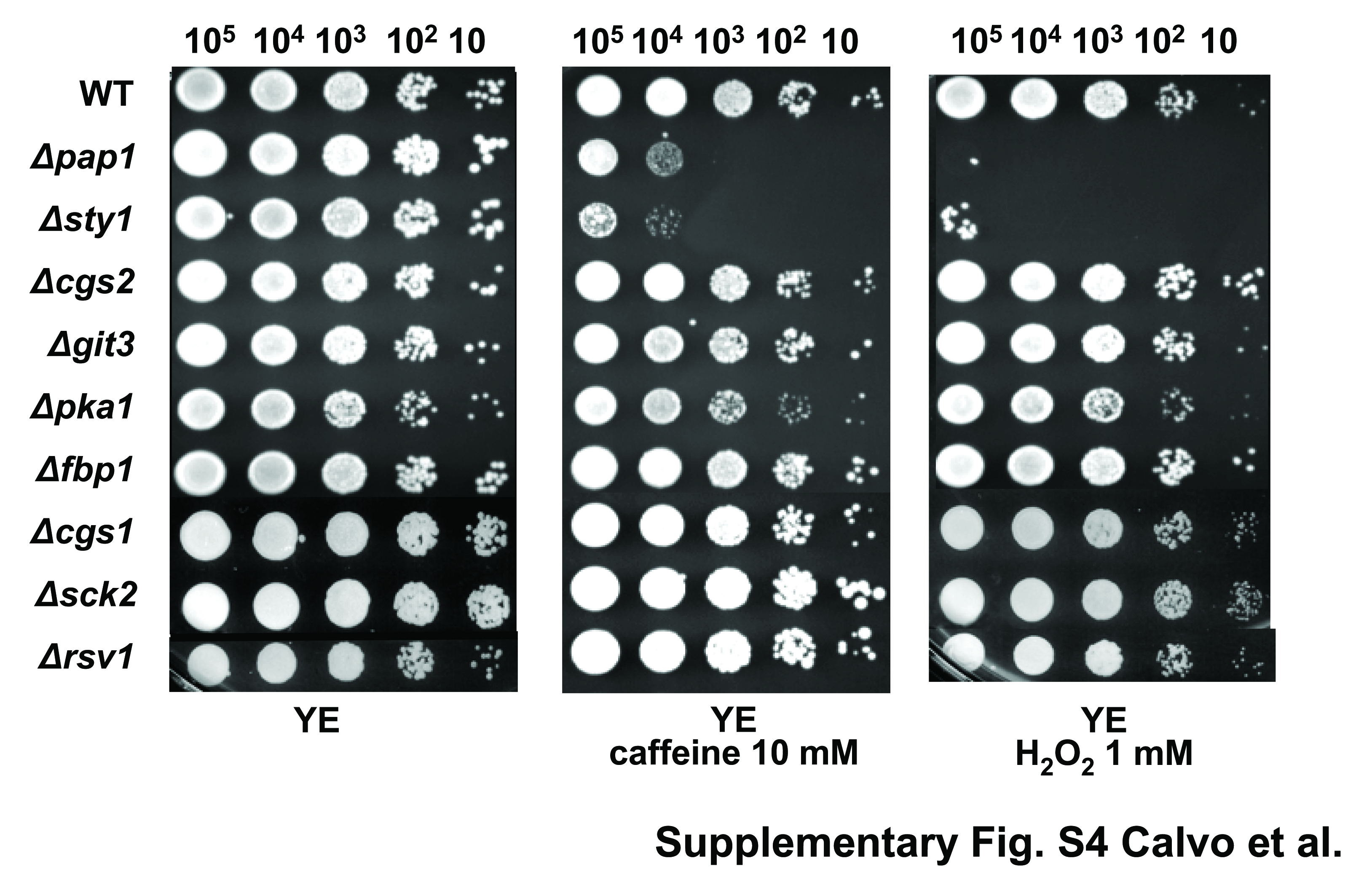

Supplement: Figure S4 — The protein kinase A pathway is not required for normal tolerance to caffeine. We analyzed by sequential spotting (as described in Fig. 2D) the survival to caffeine or H2O2 exposure at the indicated concentrations of the deletion collection strains 666 (WT), Δpap1, Δsty1, Δcgs2, Δgit3, Δpka1, Δfbp1, Δcgs1, Δsck2 and Δrsv1. (3.77 MB TIF) [file pone.0006619.s005.tif]

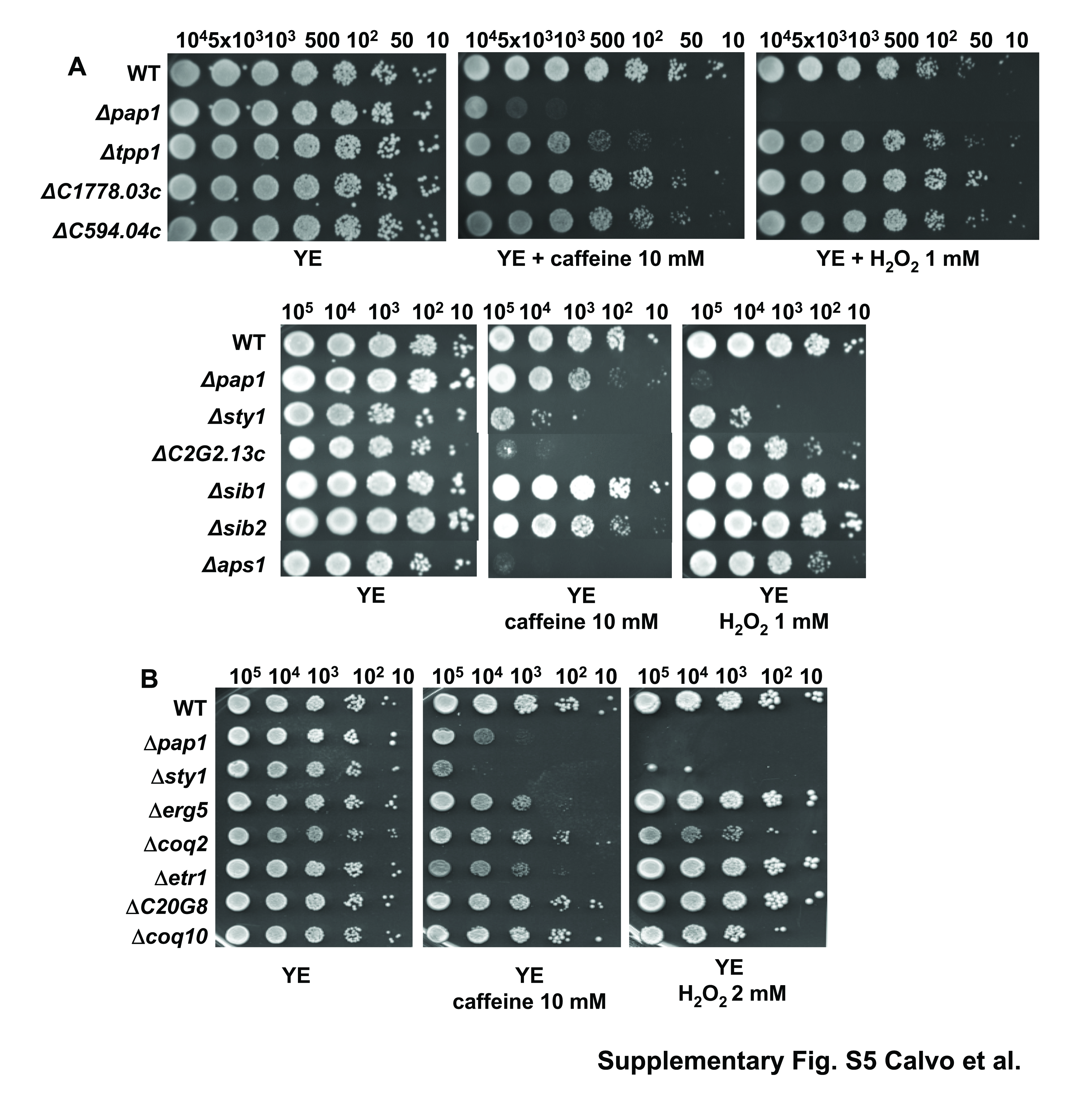

Supplement: Figure S5 — Several genes coding for enzymes related to metabolic pathways (A) and for mitochondrial components (B) are required for normal tolerance to caffeine. We analyzed by sequential spotting (as described in Fig. 2D) the survival to caffeine or H2O2 exposure at the indicated concentrations of the deletion collection strains 666 (WT), Δpap1, Δtpp1, ΔC1778.03c, ΔC594.04c, Δsty1, ΔC2G2.13c, Δsib1, Δsib2, Δaps1, Δerg5, Δcoq2, Δetr1, ΔC20G8 and Δcoq10. (6.58 MB TIF) [file pone.0006619.s006.tif]

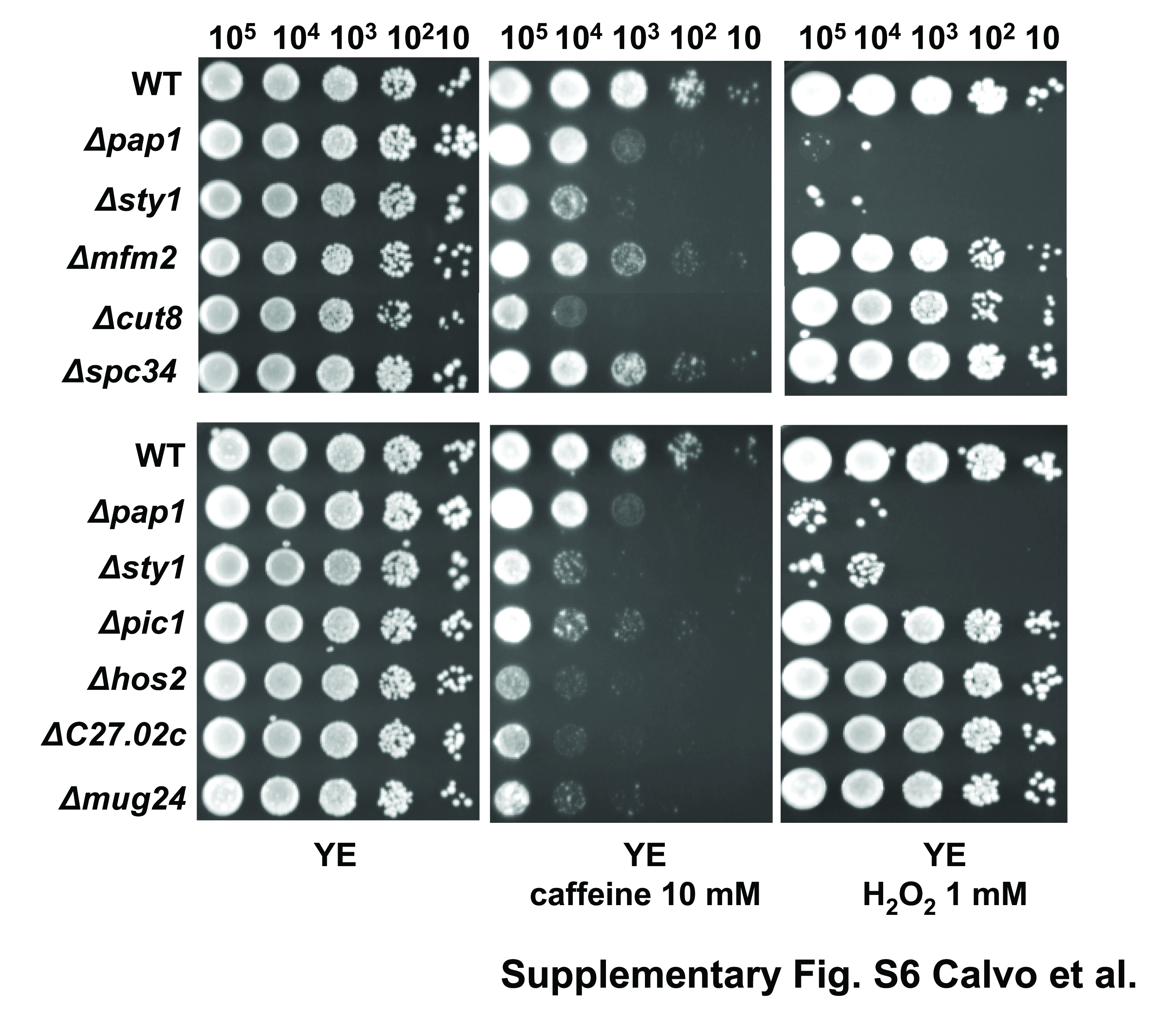

Supplement: Figure S6 — Several regulators of the mitotic or meiotic cell cycles are required for normal tolerance to caffeine. We analyzed by sequential spotting (as described in Fig. 2D) the survival to caffeine or H2O2 exposure at the indicated concentrations of the deletion collection strains 666 (WT), Δpap1, Δsty1, Δmfm2, Δcut8, Δspc34, Δpic1, Δhos2, ΔC27.02c and Δmug24. (4.31 MB TIF) [file pone.0006619.s007.tif]
